# CT-Derived Quantitative Image Features Predict Neoadjuvant Treatment Response in Adenocarcinoma of the Gastroesophageal Junction with High Accuracy

**DOI:** 10.3390/cancers17020216

**Published:** 2025-01-10

**Authors:** Markus Graf, Sebastian Ziegelmayer, Stefan Reischl, Yannick Teumer, Florian T. Gassert, Alexander W. Marka, Philipp Raffler, Jeannine Bachmann, Marcus Makowski, Daniel Reim, Fabian Lohöfer, Egon Burian, Rickmer Braren

**Affiliations:** 1 Department of Diagnostic and Interventional Radiology, School of Medicine & Klinikum Rechts der Isar, Technical University of Munich, 81675 Munich, Germany; s.ziegelmayer@tum.de (S.Z.); stefan.reischl@tum.de (S.R.); florian.gassert@tum.de (F.T.G.); alexander.marka@tum.de (A.W.M.); philipp.raffler@tum.de (P.R.); marcus.makowski@tum.de (M.M.); fabian.lohoefer@tum.de (F.L.); r.braren@tum.de (R.B.); 2 Department of Medicine II, Ulm University Medical Center, 89081 Ulm, Germany; yannick.teumer@uni-ulm.de; 3 Department of Surgery, School of Medicine & Klinikum Rechts der Isar, Technical University of Munich, 81675 Munich, Germany; jeannine.bachmann@tum.de (J.B.); daniel.reim@tum.de (D.R.); 4 Diagnostic and Interventional Radiology, University Hospital Zurich, University Zurich, 8006 Zurich, Switzerland; egon.burian@usz.ch; 5 Faculty of Medicine, University of Zurich, 8006 Zurich, Switzerland; 6 German Cancer Consortium (DKTK, Partner Site Munich), German Cancer Research Center (DKFZ), 69120 Heidelberg, Germany

**Keywords:** adenocarcinoma of the GEJ, computed tomography, neoadjuvant therapy, cancer

## Abstract

Background: The purpose of this retrospective study was to evaluate the value of contrast-enhanced computed tomography (CE-CT) image features at baseline and after neoadjuvant chemotherapy in predicting histopathological response in patients with adenocarcinoma of the gastroesophageal junction (GEJ). Methods: A total of 105 patients with a diagnosis of adenocarcinoma of the GEJ were examined by CE-CT at baseline and preoperatively after neoadjuvant chemotherapy. All patients underwent surgical resection. Histopathological parameters and tumor regression grading according to Becker et al. were collected in 93 patients. Line profiles of the primary tumor area in baseline and preoperative CE-CT were generated using ImageJ. Maximum tumor density and tumor-to-wall density delta were calculated and correlated with the histopathological tumor response. In addition, tumor response was assessed according to standard RECIST measurements in all patients and by endoscopy in 72 patients. Results: Baseline and change in baseline to preoperative CE-CT parameters showed no significant differences between responders (Becker grade 1a, 1b) and non-responders (Becker grade 2, 3). After neoadjuvant therapy, responders and non-responders showed significant differences in maximum density and tumor-to-wall density delta values. Line profile measurements showed excellent inter-rater agreement. In comparison, neither RECIST nor endoscopy showed significant differences between these groups. Conclusions: Posttreatment CE-CT can predict histopathological therapy response to neoadjuvant treatment in adenocarcinoma of GEJ patients with high accuracy and thus may improve patient management.

## Simple Summary

Effective assessment of treatment response is essential for optimizing outcomes in cancer patients. This study examines how specific features derived from CT scans can accurately predict the effectiveness of preoperative chemotherapy in patients with carcinoma of the gastroesophageal junction. We found measurable differences in imaging data between patients with significant tumor regression and those with limited or no response. These findings highlight the potential for advanced imaging to enhance personalized treatment strategies and improve overall patient management. This research offers a new approach to personalized cancer care.

## 1. Introduction

Adenocarcinoma of the GEJ is a distinct tumor entity that was classified into three types by Siewert et al. based on their location within an aboral and oral distance of five centimeters to the esophagogastric junction, defined as the Z-line. A marked increase in adenocarcinomas of the GEJ in the Western world has been noted in recent decades [1]. While the only curative treatment is an extensive tumor resection, neoadjuvant chemoradiation or chemotherapy are recommended for all ≥ cT2 tumors in the current NCCN guidelines [2]. For neoadjuvant treatment, a prolongation of overall and progression-free survival has been demonstrated [3,4]. Currently, the tumor response to neoadjuvant treatment is assessed by histopathology [5]. Several tumor regression grading (TRG) systems have been defined. The most common is the Becker and Mandard score, which measures vital residual tumor cells. No difference in the prognostic significance of the two systems can be detected [6,7].

The choice of maximum tumor density as a metric in our study was based on its ability to reflect the overall cellularity and structure of the tumor. Higher densities are often associated with viable tumor tissue, while lower densities may indicate necrosis or response to therapy [8]. Changes in tumor density, such as those induced by chemotherapy, are critical indicators of treatment efficacy and provide non-invasive methods to evaluate tumor response [9,10].

According to Becker et al., histopathological regression grading is defined as 1a: no residual tumor, 1b: <10% residual tumor cells, 2: 10–50% residual tumor, and 3: >50% residual tumor in the primary tumor bed [11]. Although patients with a major pathological response (i.e., responders with TRG of Becker grade 1a and 1b) show significantly improved overall survival, the identification of non- or moderate responders is of great importance, as they are exposed to potential chemotoxicity and may benefit from primary surgery [12] or alternative neoadjuvant regimens. Therefore, tools to identify the projected response to neoadjuvant therapy are of high interest. The Response Evaluation Criteria in Solid Tumors (RECIST) are a guideline for clinical tumor response assessment based on cross-sectional imaging [10]. For the majority of solid tumors, there is no established standard for evaluating the response to neoadjuvant therapy. The literature recommends the imaging modality of clinical staging, as it allows the greatest comparability [13], and in cases of adenocarcinoma of the GEJ, CE-CT and endoscopy are routinely performed.

RECIST are only conditionally suitable for evaluating treatment response, as measurements can only be determined to a limited extent due to (semi)circular tumor growth and wall morphology with fibrotic changes after neoadjuvant chemotherapy [14]. Therefore, the primary tumor often is not classified as a target lesion in patients with adenocarcinoma of the GEJ. Endoscopy can reliably detect changes in the tumor surface, and expandability in response to air insufflation can be used as an indirect sign of tumor regression. However, in-depth assessment of the tumor microenvironment goes beyond imaging-based characterization and includes cellular investigations as well as genetic analyses [5,15,16].

Therefore, the primary aim of our study was to analyze the correlation between attenuation-based CE-CT parameters of tumors at baseline and after neoadjuvant treatment as a possible surrogate of TRG. The findings were compared to standard assessment of treatment response based on RECIST and endoscopy.

## 2. Materials and Methods

### 2.1. Patient Collective and Image Acquisition

This study was designed as a single-center retrospective cohort study. Data collection, processing, and analysis were approved by the institutional review board (protocol 180/17S) and informed consent was waived. Patients presenting with adenocarcinoma of the GEJ at our hospital between 01/01/2009 and 01/08/2022 were analyzed for eligibility. Inclusion criteria were (1) neoadjuvant chemotherapy or chemoradiation therapy followed by tumor resection or palliative therapy regimen, (2) histopathologically proven adenocarcinoma of the GEJ, and (3) availability of CE-CT at baseline and preoperatively after neoadjuvant treatment. Exclusion criteria were (1) histopathologically proven squamous cell carcinoma and (2) insufficient quality of CE-CT (e.g., heavy motion and breathing artifacts) or (3) missing follow-up dataset.

All patients underwent a standardized CT protocol using either the Iqon Spectral CT scanner or the iCT scanner (both Philips, Best, The Netherlands). Images were acquired in arterial and venous contrast phase after intravenous application of iodine contrast agent (Ultravist^®^-370, Bayer, 80 mL, 3.5 mL/s) and bolus tracking with an ROI placed in the aorta. A soft tissue reconstruction kernel was used for multi-planar reconstruction.

In addition, 71 patients received preoperative endoscopy-based staging and were classed as progressive, stable, or regressive. Patients were classified as “responders” when showing major TRG (Becker regression grade 1a and 1b) or “non-responders” (Becker regression grade 2 and 3) based on routine pathological reports. The 12 patients in palliative settings were included in the analysis.

### 2.2. Image Analysis

**Figure 1:**
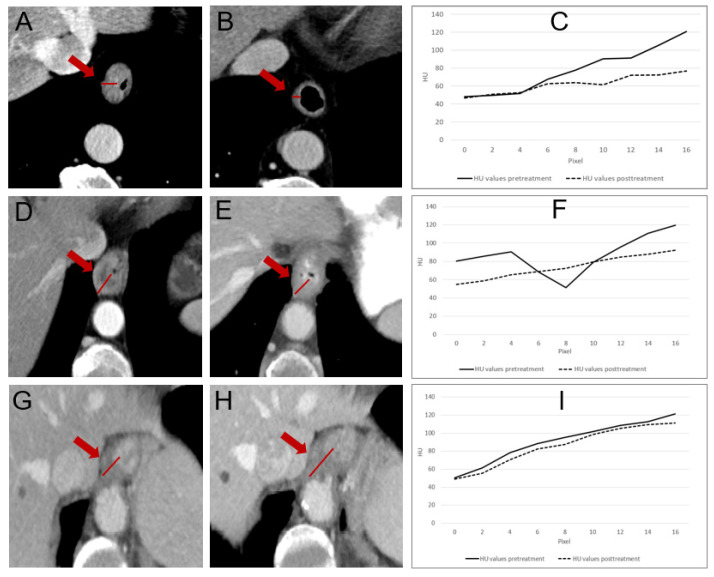
(**A**) Conventional iodine-enhanced CT in a patient diagnosed with type I adenocarcinoma of the GEJ. A line profile was set in the tumor region, with maximum HU value of 120.69 and density delta value of 69.05. (**B**) The same patient’s CE-CT after neoadjuvant treatment (grading of regression 1b). A line profile was again set in the prior tumor area, showing a maximum HU value of 76.65 and a density delta value of 24.19. (**C**) Plot profile showing a significant posttreatment HU value drop. (**D**,**E**) Iodine-enhanced CT scans in a patient with type I adenocarcinoma of the GEJ (grading of regression 2). Maximum pretreatment HU value is 119.87, maximum posttreatment HU value is 91.25. (**F**) Corresponding plot profile. (**G**,**H**) Line profiles in the tumor region of another non-responding type I adenocarcinoma of the GEJ patient (grading of regression 3). The maximum pretreatment HU value in **G** is 121.48 and posttreatment HU in (**H**) is 112.36. (**I**) Plot profile with no significant drop after neoadjuvant therapy.

**Figure 2:**
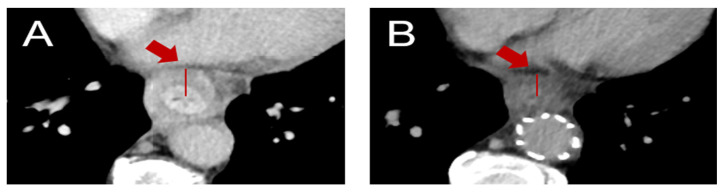
(**A**) Conventional iodine-enhanced CT in a patient diagnosed with type I adenocarcinoma of the GEJ. A line profile was set again in the tumor region, with maximum HU value of 129.75 and density delta value of 94.57. (**B**) The same patient’s CE-CT after neoadjuvant treatment (grading of regression 1a). A line profile was again set in the prior tumor area, with maximum HU value of 84.78 and density delta value of 39.53.

A line profile using the open-source software ImageJ^®^ (Version 1.53) was manually placed in an axial image slice upon visual inspection, covering the highest-density area of the tumor and normal esophageal wall. For consistency in comparison, the density profiles were normalized to a common scale (0–16 pixels), regardless of the actual length of the line profiles in the images. For consistency in comparison, the density profiles were normalized to a common scale (0–16 pixels), regardless of the actual length of the line profiles in the images. This normalization process allowed us to compare density changes across tumors on a standardized scale. Explanatory visualizations are shown in Figure 1 and Figure 2.

Maximum tumor density and tumor-to-wall density delta were calculated from these line profiles of baseline and preoperative (after successful neoadjuvant treatment) CE-CT. The tumor-to-wall density delta was chosen to capture the contrast between the tumor and surrounding normal tissue, providing a relative measure that may be more robust to variations in imaging conditions and patient factors. A region of interest with a diameter of 5 mm was placed in the thoracoabdominal aorta, and each metric was normalized based on the metric/aortic density value to account for differences in contrast. To address inter-reader agreement, 40 cases were measured by a second reader (S.Z.) and the intraclass correlation coefficient was calculated. In addition, preoperative CE-CT was classified according to RECIST 1.1 (Response Evaluation Criteria in Solid Tumors). Due to the irregular morphology of the primary tumor in gastroesophageal junction (GEJ) adenocarcinoma, the primary tumor is often not classified as a target lesion. The RECIST in this study were based on measurable changes in primary tumor diameter and morphology: Partial response (PR): At least a 30% decrease in the sum of target lesion diameters, relative to baseline sum diameters. Stable disease (SD): Neither sufficient shrinkage to qualify for PR nor sufficient increase to qualify for progressive disease, using as reference the smallest sum diameter during the study. Progressive disease (PD): At least a 20% increase in the sum of target lesion diameters, using the smallest sum on study as reference, or the appearance of one or more new lesions. The classification was performed by an experienced radiologist (M.G.). Furthermore, endoscopy-based restaging classification was collected from electronic health records. Each CE-CT-derived feature and staging was analyzed for significant differences between responders and non-responders. For CE-CT-derived features, in cases of a significant difference between responders and non-responders, the respective feature was further tested for differences between the individual regression grades according to Becker.

### 2.3. Statistical Analysis

All statistical analyses were performed by S.Z. using Python (Version 3.8) with SciPy (version 1.10.1). Categorical variables are expressed as frequencies and percentages, and continuous variables are expressed as mean ± standard deviation. The tested data were visually evaluated for normal distribution. Two-sided *t*-tests were applied for the numeric parameters, and the Mann–Whitney U-test was used for categorical parameters. Intraclass correlation coefficients were calculated using the pingouin package. A *p*-value < 0.05 was considered statistically significant. In addition, receiver operating characteristic (ROC) curves, sensitivity, and specificity were calculated for the identified density values. ROC curves were generated using the scikit-learn library in Python. The area under the curve (AUC) was determined to evaluate the discriminative power of the density values.

## 3. Results

### 3.1. Study Population

**Figure 3:**
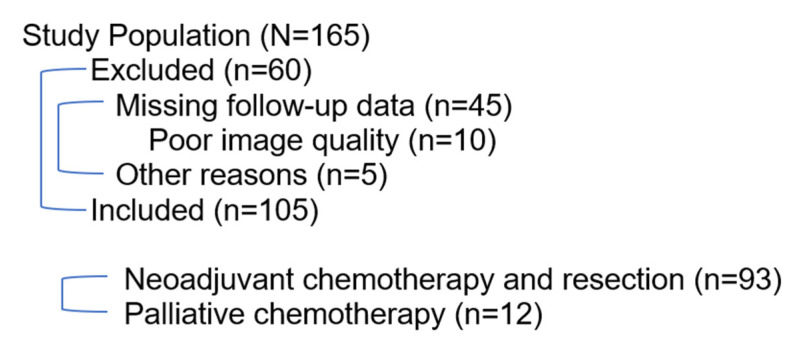
Accrual flowchart.

Overall, 165 patients diagnosed with adenocarcinoma of the GEJ between January 2009 and August 2022 at our tertiary institution were screened for eligibility. Of these, 60 were excluded due to missing follow up data. Average age at diagnosis was 67 ± 11 years (IQR: 60–76 years; range 26–85 years). The median overall survival in the cohort was 33 months. In sum, 93 patients received chemotherapy followed by tumor resection with curative intent and 12 patients received chemotherapy with palliative intent. Chemotherapy included various regimens, primarily based on fluorouracil, leucovorin, oxaliplatin, and docetaxel (FLOT) combined with immunotherapy (*n* = 71) or folinic acid (leucovorin), fluorouracil (5-FU), and oxaliplatin (FOLFOX) (*n* = 14). Histopathological tumor category (ypT) was divided into ypT0 (*n* = 18), ypT1 (*n* = 12), ypT2 (*n* = 15), ypT3 (*n* = 48), and ypT4 (*n* = 0). Specimens showed an even distribution of Becker regression grades (1a: *n* = 22, 1b: *n* = 23, 2: *n* = 25 and 3: *n* = 23). An accrual flowchart and study population characteristics are shown in Figure 3 and Table 1.

### 3.2. Imaging and Correlation of the Histopathological Regression Grade

**Figure 4:**
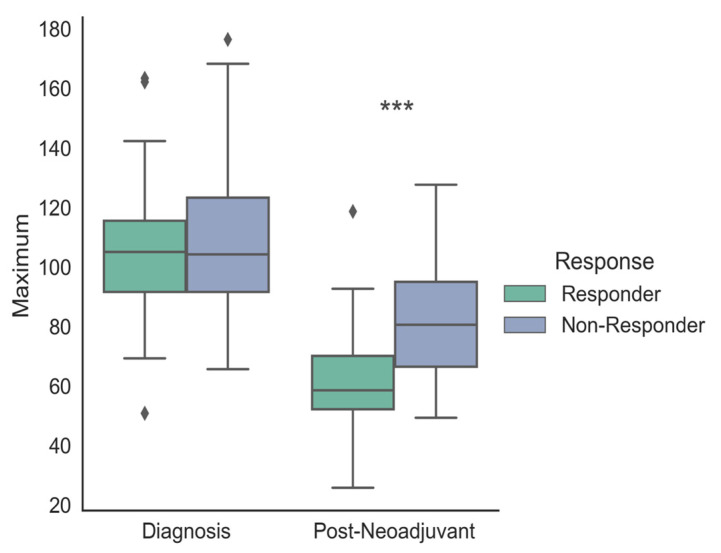
Comparison of delta HU values within the line profile set intralesionally in the tumor area after neoadjuvant chemotherapy of different histopathological regression grades (Ia–III) and palliatively managed patients. Diamonds (♦) represent outliers, i.e., data points that fall outside 1.5 times the interquartile range (IQR) above the upper quartile or below the lower quartile. ***: *p* < 0.001.

Preoperative (posttreatment) CE-CT image analysis revealed significant differences in tumor-to-wall density delta between responders and non-responders (Figure 4; 29.44 ± 10.44 and 62.47 ± 22.29, *p* < 0.001).

**Figure 5:**
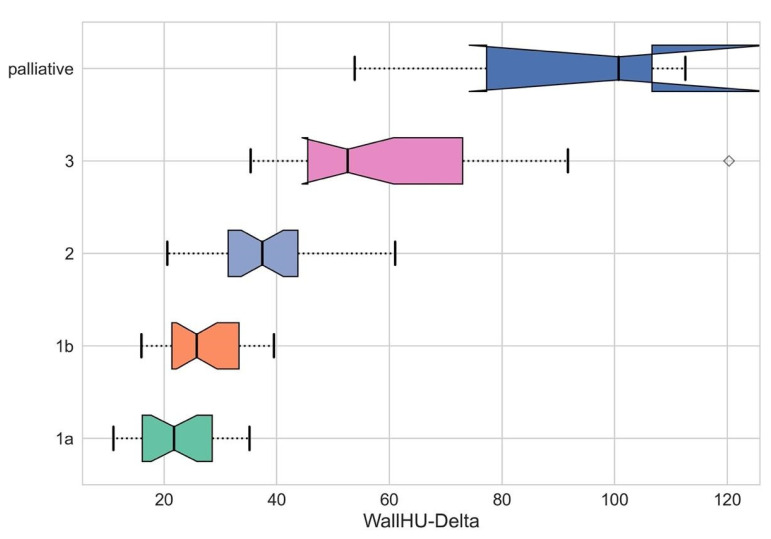
Findings of max. density value and density delta in patients defined as histopathological responders (<10% tumor cells, grade 1a and 1b according to Becker et al.) and non-responders (>10% tumor cells according to Becker et al.).

**Figure 6:**
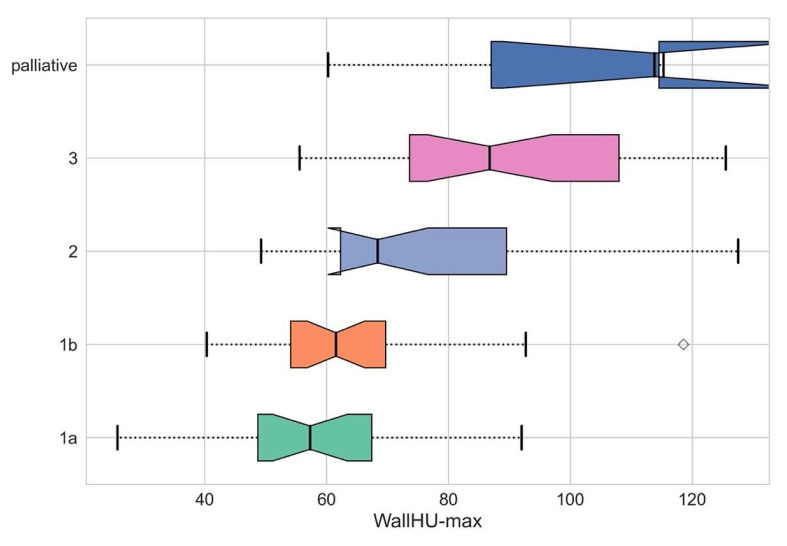
Comparison of maximum HU values within the tumor area of the different degrees of regression according to Becker et al.

Similarly, significant differences in maximum tumor density of responders and non-responders were noted (Figure 4; 61.07 ± 16.73 and 83.05 ± 21.66, *p* < 0.001). These differences in tumor-to-wall density delta and maximum tumor density remained significant even in the more granular comparison of the individual Becker grades (tumor-to-wall density delta: 1a: 22.27 ± 3.94, 1b: 27.18 ± 5.54, 2: 38.6 ± 9.55 and 3: 59.63 ± 3.73, *p* < 0.001; maximum tumor density: 1a: 57.96 ± 6.42, 1b: 63.74 ± 3.15, 2: 77.89 ± 2.37 and 3: 90.02 ± 12.00). The highest values for both parameters were seen the palliative (71.35 ± 10.27 and 94.59 ± 4.28) and progressive disease patients (*n* = 3; 89.02 ± 25.36 and 96.45 ± 25.62) (Figure 5 and Figure 6).

**Figure 7:**
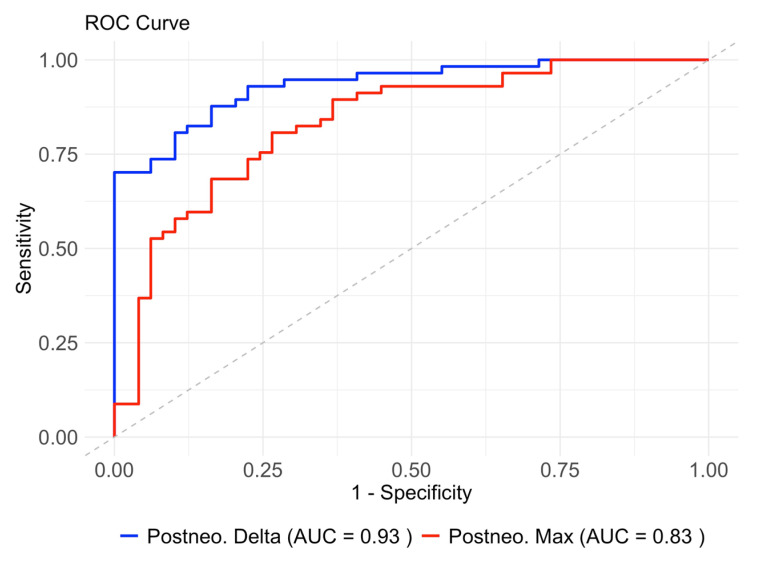
Receiver operating characteristic (ROC) curves for maximum density and density delta derived from post-treatment CT data. The ROC curve for maximum density shows an area under the curve (AUC) of 0.83 with an optimal threshold (66.50), resulting in a sensitivity of 0.81 and a specificity of 0.73. The ROC curve for delta density shows a higher AUC of 0.93 with an optimal threshold (34.17), resulting in a sensitivity of 0.88 and a specificity of 0.84. The dashed diagonal line represents the line of no discrimination (AUC = 0.50).

The ROC analysis for posttreatment parameters showed an AUC of 0.83 for maximum density and 0.93 for density delta, indicating a higher discriminative power for density delta (Figure 7).

The optimal threshold for density delta resulted in a sensitivity of 0.88 and a specificity of 0.84, while the optimal threshold for maximum density resulted in a sensitivity of 0.81 and a specificity of 0.73.

**Figure 8:**
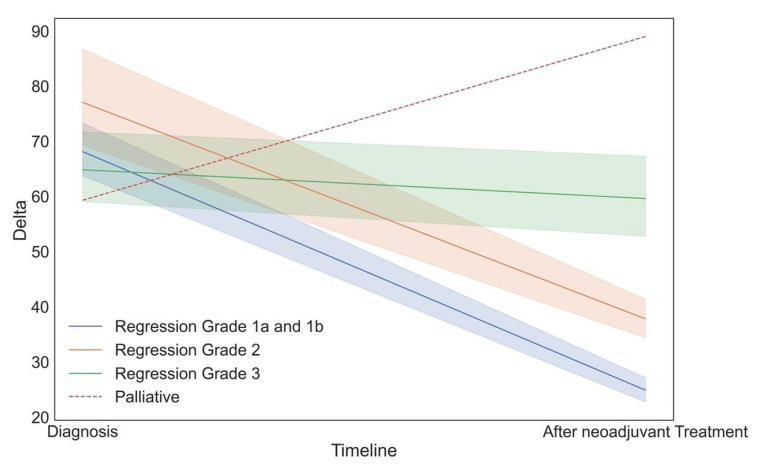
Delta HU development after neoadjuvant treatment subdivided into the different histopathological degrees of regression and for palliatively managed patients. The shaded areas represent one standard deviation (SD).

In contrast, neither baseline CE-CT tumor-to-wall density delta nor maximum tumor density differentiated responders from non-responders (71.27 ± 20.61 versus 64.32 ± 17.45, *p* < 0.001 and 105.45 ± 21.77 versus 108.45 ± 25.69, *p* < 0.001). The same was true for the changes in these parameters from baseline to preoperative CE-CT presented in Figure 8.

The evaluation of the ICC showed excellent inter-rater agreement for both maximum tumor density and tumor-to-wall density delta at diagnosis and preoperatively (maximum tumor density at diagnosis ICC: 0.923, CI95 0.81, 0.97; preoperatively ICC: 0.82, CI95: 0.57, 0.93 tumor-to-wall density delta at diagnosis ICC: 0.93, CI95: 0.82, 0.97 preoperatively ICC: 0.932, CI95: 0.82, 0.97).

### 3.3. RECIST and Endoscopic Response Evaluation

To compare our results to clinical routine methodology, we applied the Response Evaluation Criteria in Solid Tumors (RECIST 1.1) to CE-CT imaging data and collected all available endoscopic report data (*n* = 72). The RECIST findings for our patient cohort are demonstrated in Figure 6. In the responder group, 24 patients were classified as partial response (PR) and 26 as stable disease (SD), whereas in the non-responder group, 18 patients were classified as PR and 37 as SD. No patient in the overall cohort was classified as progressive disease (PD). According to endoscopic re-evaluation after neoadjuvant chemotherapy, 5 patients in the responder group had stable and 30 had regressive findings. In contrast, a single patient in the non-responder group had a progressive finding, while 15 patients were classified stable and 21 patients exhibited signs of regressive disease.

## 4. Discussion

In our study, we identified preoperative (posttreatment) CE-CT-derived image feature tumor-to-wall density delta and maximum tumor density as excellent discriminators of histopathological responders and non-responders in patients with adenocarcinoma of the GEJ undergoing neoadjuvant chemotherapy. ROC analysis confirmed these findings, with AUC values of 0.83 for maximum density and 0.93 for density delta, indicating higher discriminative power for density delta. The tumor-to-wall density delta parameter even enabled a significant separation of all four individual histopathological Becker regression grades. The strong correlation between these CT-derived features and TRG underscores their relevance in clinical practice. In contrast, baseline CE-CT parameters did not show a significant correlation with TRG. Moreover, both, RECIST and endoscopic staging failed to reliably separate responders from non-responders. The superior performance of the tumor-to-wall density delta metric compared to RECIST and endoscopic assessment highlights the importance of relative density measures. RECIST, which are primarily based on tumor size, often fail to accurately assess treatment response due to their inability to account for changes in tumor composition and density. Studies have shown that density-based metrics can provide a more robust and accurate assessment of treatment response, particularly in gastrointestinal cancers where traditional size-based criteria are less effective [10,17].

Although neoadjuvant therapy is widely established for GEJ cancer, there are no clear recommendations for chemotherapy regimens and adjunctive radiotherapy [18]. Furthermore, a relevant percentage of patients do not respond sufficiently to the neoadjuvant therapy or even show tumor progression. These patients are exposed to side effects of chemotherapy without benefit and a potentially curative resection may be delayed, emphasizing the importance of neoadjuvant response evaluation [19]. Our findings underscore the importance of accurate assessment of tumor response. As highlighted in recent studies, this can lead to improved patient outcomes when performed in high-volume, specialized centers [20].

Early-response evaluation using PET-CT with fluorodeoxyglucose (FDG) has been shown to be effective in patients with adenocarcinoma of the GEJ undergoing neoadjuvant treatment. Furthermore, this crossover trial suggests that PET-CT can be used to stratify non-responders to alternative chemotherapy regimen achieving higher histopathological response rates [19]. As shown by Schneider et al., PET-CT can identify non-responders, but shows no apparent correlation with the overall pathological response [21].

However, histopathological regression grade (TGR) is better suited to evaluating tumor response, as it correlates significantly with complete tumor resection status, histopathological tumor category after neoadjuvant treatment (ypT), lymph node involvement, lymphatic vessel invasion, and overall patient survival [22]. In comparison, non-responders, characterized by >10% of remaining tumor cells, had greater postoperative pulmonary morbidity, a greater 30-day mortality rate, and a dismal survival rate compared to histopathological responders. Another recent study has shown that Hounsfield unit change measured in CT after neoadjuvant chemotherapy can separate responders and non-responders [23]. Cihan et al. observed that a greater change in HU was associated with a worse pathological response in gastric adenocarcinoma, especially in T4 tumors. In contrast, our study focused on GEJ adenocarcinoma stages T0–T3 and found that a greater change in tumor-to-wall density delta was associated with a better pathological response. Cihan et al. measured total HU changes, while our study used tumor-to-wall density delta. These differences in tumor type, stage, and measurement metrics may account for the observed discrepancies.

Our study shows that the post-neoadjuvant HU delta between the tumor and normal esophageal tissue can additionally separate all TGR grades, which holds the potential to allow a more individualized approach in therapy modification, as a binary classification may neglect relevant prognostic differences [24]. Although PET-CT examination is recommended as initial staging, there is no standard for therapy evaluation to date and contrast-enhanced CT would be a feasible alternative with comparably lower radiation exposure [2]. Furthermore, if a PET-CT examination is recommended as the standard of care for therapy evaluation, HU delta in combination with tracer uptake may provide a more accurate prediction of response, particularly in tumors that are not FDG-avid.

**Figure 9:**
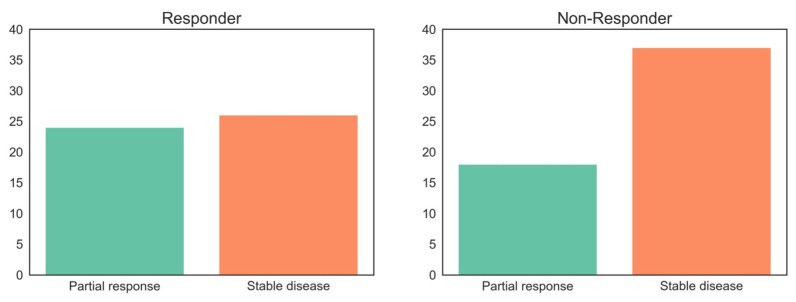
Correlation of therapy response according to RECIST 1.1 and histopathological response degree according to Becker et al.

Beyond what we could line out, that the current radiological evaluation system with the classification of response evaluation criteria in solid tumors (RECIST 1.1) is insufficient for detecting neoadjuvant therapy responders (Figure 9).

**Figure 10:**
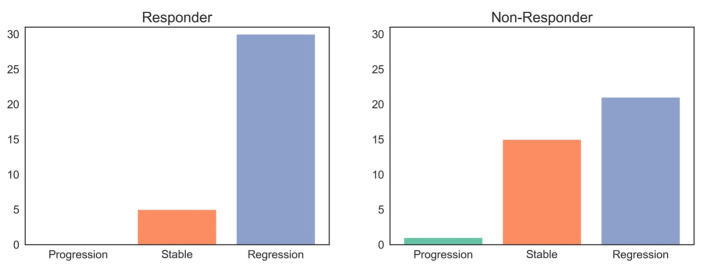
Correlation of the esophagogastroduodenoscopy findings and tumor regression grading.

In addition to that, the esophagogastroduodenoscopy findings in our cohort show that endoscopic correlation of therapy success can be difficult and show inadequate results, as a high percentage of responders are defined as stable disease and a relevant number of non-responders are classified as regressive (Figure 10).

Despite promising results, our study shows several limitations that need to be addressed. The retrospective design and small cohort limit broad generalizability, which makes further prospective validation necessary. In our study, preoperative CT scans were used for correlation with the TGR. For a modification of the neoadjuvant therapy depending on tumor response, an evaluation at an earlier time point, for example, after the first cycle, is necessary and needs to be investigated in follow-up studies. As a proof of concept, we were able to show a strong correlation between the HU delta and the TGR for preoperative scans (Figure 4). As with all manually defined metrics, the line profile can be influenced by the subjectivity of the observer. Using intraclass correlation coefficients, we were able to show excellent inter-rater agreement, indicating measurement reproducibility. Additionally, attenuation-dependent parameters appear to contain relevant information regarding treatment response that are independent of the measurement method, indicating that changes in attenuation are a possible surrogate parameter for tumor perfusion and/or tumor viability. To address the potential observer bias associated with manual line profile placement, future studies could explore automated image analysis techniques, such as artificial intelligence (AI)-based segmentation tools.

## 5. Conclusions

To conclude, tumor-to-wall intensity delta allows the prediction of the histopathological response to neoadjuvant treatment in adenocarcinomas of the GEJ beyond a binary responder and non-responder classification. Our findings hold the potential to allow an individualized modification of neoadjuvant treatment strategies in adenocarcinomas of the GEJ.

## Figures and Tables

**Table 1:** Study population characteristics.

Characteristics	Total (*n* = 105)	Neoadjuvant Therapy (*n* = 93)	Palliative Therapy (*n* = 12)
Age (median ± SD)	67 ± 11	66 ± 12	69 ± 9
Gender Male Female	78 27	70 23	8 4
Histology Adenocarcinoma yT0 yT1 yT2 yT3 yT4	105	93 18 12 15 48 0	12
Becker Regression Grade 1a 1b 2 3		22 23 25 23	
Chemotherapy Regimen FLOT FOLFOX	71 14	63 12	8 2

## Data Availability

The data that support the findings of this study are available from the corresponding author (M.G.) upon reasonable request.
